# Recent Understandings Toward Coronavirus Disease 2019 (COVID-19): From Bench to Bedside

**DOI:** 10.3389/fcell.2020.00476

**Published:** 2020-06-09

**Authors:** Jie Yu, Peiwei Chai, Shengfang Ge, Xianqun Fan

**Affiliations:** Department of Ophthalmology, Shanghai Key Laboratory of Orbital Diseases and Ocular Oncology, Nineth People's Hospital, Shanghai JiaoTong University School of Medicine, Shanghai, China

**Keywords:** COVID-19, SARS-CoV-2, 2019-nCoV, health emergency, review

## Abstract

In late December 2019, an unprecedented outbreak of coronavirus disease 2019 (COVID-19) caused by SARS coronavirus 2 (SARS-CoV-2) (previously named 2019-nCoV) in Wuhan became the most challenging health emergency. Since its rapid spread in China and many other countries, the World Health Organization (WHO) declared COVID-19 a public health emergency of international concern (PHEIC) on 30th January 2020 and a pandemic on 11th March 2020. Thousands of people have died, and there are currently no vaccines or specific antiviral drugs for COVID-19. Therefore, it is critical to have a comprehensive understanding of the virus. In this review, we highlight the etiology, epidemiology, pathogenesis and pathology, clinical characteristics, diagnosis, clinical management, prognosis, infection control and prevention of COVID-19 based on recent studies.

## Introduction

In late December 2019, the rapid outbreak of coronavirus disease 2019 (COVID-19) caused by SARS coronavirus 2 (SARS-CoV-2) (previously named 2019-nCoV) in Wuhan, Hubei Province, has attracted worldwide attention (Wu F. et al., 2020; Zhu et al., 2020). Most of the initial cases of COVID-19 were linked to exposure to the Huanan Seafood Wholesale Market, where live animals, including bats and snakes, are traded. Though the market was closed soon, many people who had close contact with a patient or a history of travel to Wuhan were infected with the SARS-CoV-2 and had symptoms of fever, cough, and dyspnea (Huang et al., 2020; Lauer et al., 2020). Since its rapid spread in China and many other countries, COVID-19 has been declared a public health emergency of international concern (PHEIC) by the World Health Organization (WHO) on 30th January 2020. Later, the WHO formally named the disease COVID-19 on February 11, 2020, and the Coronavirus Study Group (CSG) of the International Committee on Virus Taxonomy named the virus SARS-CoV-2 instead of 2019-nCoV on the same day (Gorbalenya et al., 2020); however, this new name was opposed by a group of virologists in China for its misleadingness and confusion (Jiang et al., 2020).

Coronaviruses (CoVs), which were first identified in the 1960s, are common pathogens in humans and animals such as birds, bats, and snakes and belong to the order Nidovirales, family Coronaviridae, and subfamily Coronaviridae. This subfamily is classified into four genera, alphacoronavirus, betacoronavirus, gammacoronavirus, and deltacoronavirus, based on phylogenetic methods (Woo et al., 2010; Weiss and Leibowitz, 2011). CoVs are spherical, ranging from 120 to 160 nm in diameter, with 20-nm-long club-shaped projections around the outer envelope that resemble a crown or the solar corona. CoVs comprise a single-stranded positive-sense RNA and at least four structural proteins: envelope (E) protein, membrane (M) protein, nucleocapsid (N) protein, and spike (S) protein. For now, there are seven CoVs (Table 1) that are known to infect humans, including human CoV-229E, human CoV-NL63, human CoV-HKU1, human CoV-OC43, SARS-CoV, MERS-CoV, and SARS-CoV-2 (Chen Y. et al., 2020). The first four viruses are pathogens with relatively low virulence and are associated with mild disease (Su et al., 2016), while the latter three have a different pathogenicity. SARS-CoV caused the outbreak of severe acute respiratory syndrome (SARS) in China in 2002, and it caused 8,422 infections in 32 countries and 919 deaths in total (Chan-Yeung and Xu, 2003). MERS-CoV, first identified in Saudi Arabia in 2012, caused 2,494 infections and 858 deaths in 27 different countries (Memish et al., 2020).

**Table 1:** List of seven CoVs known to infect human.

**Virus**	**Genera**	**Symptoms**
Human CoV-229E	Alphacoronavirus	Mild upper respiratory disease, in rare cases can cause severe infection in infants, young children and elders
Human CoV-NL63	Alphacoronavirus	
Human CoV-OC43	Betacoronavirus	
Human CoV-HKU1	Betacoronavirus	
SARS-CoV	Betacoronavirus	Severe acute respiratory syndrome, about 10% mortality rate
MERS-CoV	Betacoronavirus	Severe acute respiratory syndrome, about 37% mortality rate
SARS-CoV	Betacoronavirus	Severe acute respiratory syndrome, about 2.3% mortality rate

To date, China is gradually bringing the outbreak under control with zero growth in the number of patients in many cities, but SARS-CoV-2 continues to spread internationally with rapid growth. Confirmed cases have been reported in more than 100 countries worldwide, and conditions in Italy, Iran, South Korea, Spain, Germany, and other countries are very serious. WHO declared the coronavirus outbreak a pandemic on 11th March 2020. Therefore, we write this review to provide an overview of COVID-19 with a focus on different aspects including etiology, epidemiology, pathogenesis and pathology, clinical characteristics, diagnosis, clinical management, prognosis, infection control, and prevention based on recent studies. We hope that governments, the public and medical workers work together globally to overcome the virus.

## Etiology

**Figure 1:**
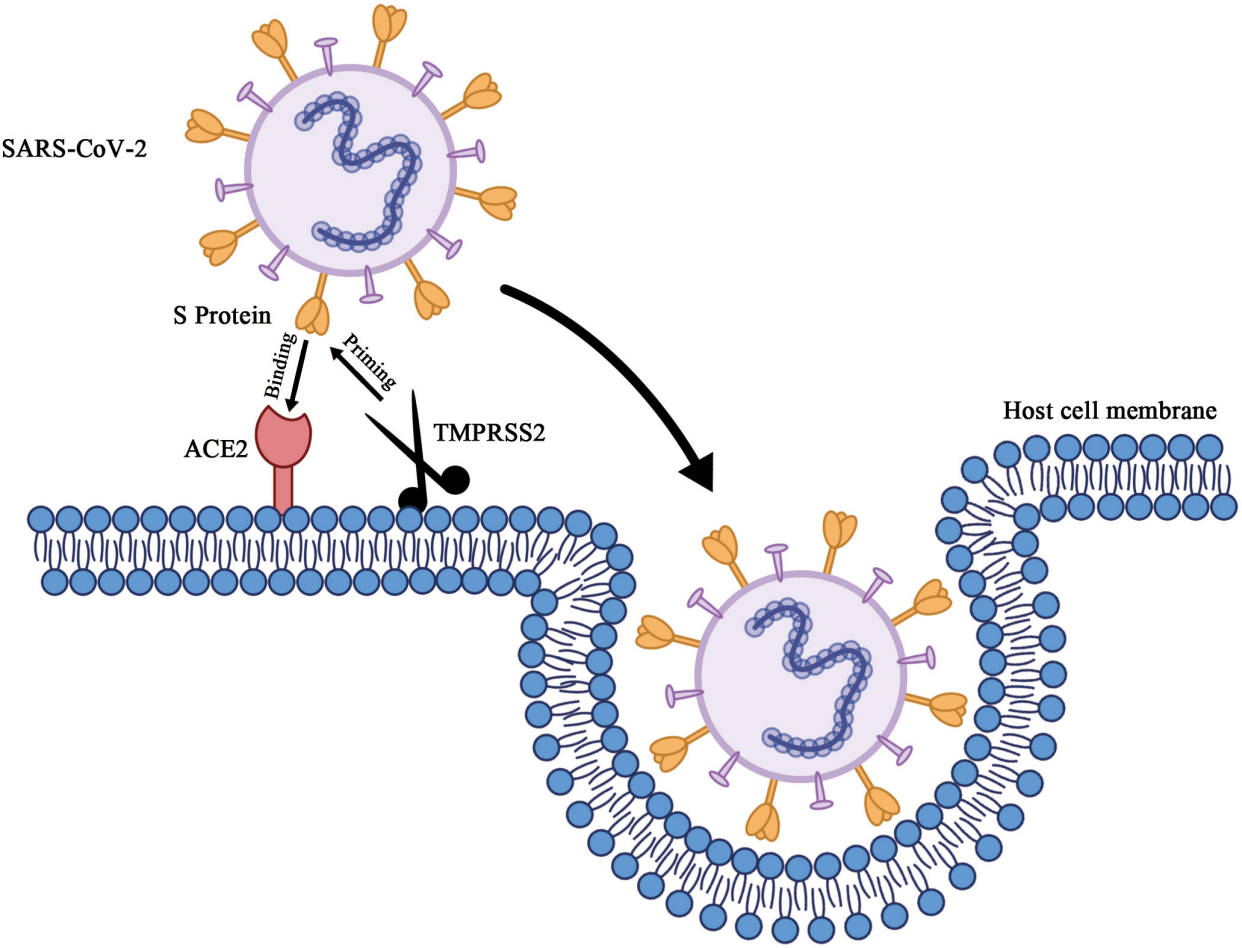
Schematic of cell entry of SARS-CoV-2. When the trimeric S proteins of SARS-CoV-2 binds to the cellular receptor ACE2, the cellular serine protease TMPRSS2, which's for S protein priming, entails S protein cleavage at the S1/S2 and the S2' site to facilitates fusion of viral envelop and host cell membranes.

SARS-CoV-2 is a betacoronavirus possessing an enveloped single-stranded RNA. It is generally spherical with some pleomorphism and ranges from 60 to 140 nm in diameter with 9- to 12-nm-long distinctive surface spikes (Zhu et al., 2020). Cell entry (Figure 1) of SARS-CoV-2 depends on these trimeric S proteins to bind to receptors. Specifically, when the S1 subunit of the S protein binds to the cellular receptor ACE2, the cellular serine protease TMPRSS2, which is for S protein priming, activates S protein cleavage at the S1/S2 and S2' sites to facilitate the fusion of the viral envelope and host cell membranes (Hoffmann et al., 2020). The crystal structure of the receptor binding domain (RBD) of sars-cov-2 S protein in complex with ACE2 has yet to be determined by Shang et al. Compared with SARS-CoV RBD, the structural features of ACE2 binding ridge in SARS-CoV-2 RBD and the change of several residues which stabilized the two viral binding hotspots on the interface of RBD-ACE2 increased the binding affinity of SARS-CoV-2 RBD and ACE2 (Shang J. et al., 2020). Similar results have also been reported by other groups (Lan et al., 2020; Wang Q. et al., 2020). Gao group and Yin group reported the cryo-electron microscopy structure of COVID-19 virus full-length RNA-dependent RNA polymerase in complex with cofactors nsp7 and nsp8 and how remdesivir binds to this target (Gao et al., 2020; Yin et al., 2020). Yuan et al. determined the structure of CR3022, a neutralizing antibody obtained from a convalescent SARS-CoV-infected patient, in complex with the RBD of the SARS-CoV-2 S protein. The antibody targets a highly conserved epitope between SARS-CoV-2 and SARS-CoV, though it binds more tightly to SARS-CoV (Yuan et al., 2020). By combining structure-based virtual and high-throughput filtering, six compounds have been found to inhibit main protease of SARS-CoV-2 (Dai et al., 2020; Jin Z. et al., 2020). Using the Immune Epitope Database and Analysis Resource, a number of specific regions with high homology to SARS-CoV virus were found in SARS-CoV-2 and parallel bioinformatics predictions identified a potential B and T cell epitope for SARS-CoV-2 (Grifoni et al., 2020). These bioinformatics or structural aspects of COVID-19 proteins information help the rapid discovery of drugs or vaccine preparation with clinical potential.

Zhou et al. reported that full-length genome sequences of SARS-CoV-2 share 79.6% sequence identity with SARS-CoV (Zhou P. et al., 2020), similar to another group's results that the virus shares ~79% sequence identity with SARS-CoV and ~50% identity with MERS-CoV (Lu R. et al., 2020). Zhou et al. also illuminated that SARS-CoV-2 shares 96% sequence identity with a bat coronavirus (BatCoV RaTG13) detected in *Rhinolophus affinis* from Yunnan Province (Zhou P. et al., 2020), with higher sequence homology than two other bat-derived SARS-like coronaviruses (bat-SL-CoVZC45 and bat-SL-CoVZXC21) found by Lu R. et al. (2020). This indicates that Chinese chrysanthemum bats are natural reservoir hosts (Zhou P. et al., 2020). Later, pangolins were found to be a possible intermediate host for SARS-CoV-2 (Lam et al., 2020; Wahba et al., 2020; Xiao et al., 2020). Andersen et al. clarified that the virus didn't emerge from a laboratory construct or a purposefully manipulated virus and they proposed two possible theories of SARS-CoV-2 origins including natural selection in an animal host before zoonotic transfer; and natural selection in humans following zoonotic transfer (Andersen et al., 2020).

The virus is sensitive to ultraviolet light and heat, and treatment at 56°C for 30 min or ethyl ether, 75% ethanol, chlorine-containing disinfectant, peracetic acid, and chloroform and other lipid solvents can effectively inactivate the virus; chlorhexidine cannot effectively inactivate the virus.

## Epidemiology

Based on previous investigations, SARS-CoV-2 is highly contagious. Patients with SARS-CoV-2 infection or even asymptomatic patients and patients during the incubation period are the main sources of infection (Rothe et al., 2020). SARS-CoV-2 is predominantly transmitted through respiratory droplets and contact (Li Q. et al., 2020). Transmission through the eyes must not be ignored since a member of the national expert panel who had eye exposure during an inspection in Wuhan was infected and had eye redness (Lu C. W. et al., 2020). Studies have shown that the virus could also be detected in stool samples, indicating its fecal–oral transmission potential (Holshue et al., 2020). In addition, there are also risks of aerosol transmission during aerosol-generating medical procedures (Wax and Christian, 2020). Vertical transmission is unlikely since an infant delivered by an infected woman was negative for COVID-19 (Li Y. et al., 2020; Stower, 2020), and amniotic fluid, cord blood, neonatal throat swab, and breastmilk samples from six pregnant patients were all negative for the virus (Chen H. et al., 2020). Lowe et al. reported the first case that parents with COVID-19 were not separated from their infant and neonatal COVID-19 testing was negative at 24 h post-delivery, suggesting rooming in post-delivery for COVID-19 positive parents is possible with viral precautions (Lowe and Bopp, 2020). Even indirect transmission may exist (Cai et al., 2020). The incubation period falls within the range of 2–14 days with a median incubation period of 5–6 days. Therefore, a 14-day period of active monitoring or quarantine was recommended for persons at risk of COVID-19 (Backer et al., 2020; Lauer et al., 2020; Linton et al., 2020; Li Q. et al., 2020).

The reproduction number (R_0_), which means the expected number of infected persons generated by one infected person on average, has been estimated by several studies. Li et al. estimated an R_0_ of ~2.2 with an epidemic doubling time of 7.4 days based on 425 confirmed cases (Li Q. et al., 2020). Wu et al. reported that R_0_ for SARS-CoV-2 was 2.68 with an epidemic doubling time of 6.4 days using data published from December 31, 2019, to January 28, 2020 (Wu J. T. et al., 2020). Crokidakis estimated that R_0_ for COVID-19 was 5.25 and the epidemic doubling time was 2.72 days in Brazil (Crokidakis, 2020). In healthcare settings, Temime et al. reported that R_0_ was 7.65 for a 170-bed rehabilitation hospital, 1.3 for an acute-care geriatric unit and 7.7 for a 100-bed nursing home (Temime et al., 2020). Furthermore, Ensser et al. estimated the daily reproduction numbers (Rt) based on new infection and death cases in the most affected European Countries and the US, which showed the strong influence of population density, behavior and cultural habits on pathogen transmission (Ensser and Ueberla, 2020; Ensser et al., 2020).

Based on all COVID-19 cases reported as of February 11, 2020, the Novel Coronavirus Pneumonia Emergency Response Epidemiology Team of the Chinese CDC described and analyzed the epidemiological characteristics of this disease. Among the 44,672 confirmed cases, most were aged between 30 and 69 years (77.8%), 51.4% were male, 22.0% were farmers or workers, and 74.7% were in Hubei Province (Epidemiology Working Group for NCIP Epidemic Response Chinese Center for Disease Control Prevention, 2020). Crowds are generally susceptible, including children (Liu et al., 2020b). Patients with cancer had a higher risk of COVID-19 (Wang and Zhang, 2020); however, Wang et al. contend that current evidence was insufficient to reach this conclusion (Xia et al., 2020). Recently, our group analyzed genetic alteration, RNA expression, and DNA methylation of ACE2 across over 30 tumors and found that overexpression of ACE2 and hypo DNA methylation of ACE2 in many in human malignancies (Chai et al., 2020).

Obviously, SARS-CoV-2 has caused far more cases than SARS-CoV. Wilder-Smith et al. offered possible explanations. Wuhan, the epicenter of COVID-19, is a major transport hub and center in China, and before the city was put in lockdown, millions of people traveled in or out due to the upcoming Spring Festival. A shortage of hospital beds resulted in many patients becoming sources of infection in the community. In addition, the transmissibility of SARS-CoV-2 might be higher than that of SARS-CoV, and asymptomatic or mildly symptomatic patients can be the sources of infection, but no known transmission occurred in such patients with SARS (Wilder-Smith et al., 2020). In addition, the affinity of the SARS-CoV-2 S protein binding to ACE2 is higher than that of the SARS-CoV S protein (Wrapp et al., 2020).

## Pathogenesis and Pathology

Zhou et al. reported the potential immunopathological mechanism by which CD4+ T lymphocytes are activated to become pathogenic T helper (Th) 1 cells and generate GM-CSF, etc., upon viral infection. A large amount of IL-6 is expressed by inflammatory CD14+CD16+ monocytes, and the inflammation accelerates. Excessive non-effective host immune responses by pathogenic T cells and inflammatory monocytes that enter the pulmonary circulation play an immune damaging role in lung pathology. Therefore, monoclonal antibodies targeting GM-CSF or IL-6 may be an effective therapy for COVID-19 patients (Zhou Y. et al., 2020).

In a 50-year-old man who died of COVID-19, a lung histological examination was characterized by bilateral diffuse alveolar damage with cellular fibromyxoid exudate, ARDS changes (the desquamation of pneumocytes and hyaline membrane formation), early-phase ARDS changes (pulmonary edema with hyaline membrane formation), interstitial mononuclear inflammatory (mainly lymphocytes) infiltrations and viral cytopathic-like changes in the intra-alveolar spaces (multinucleated syncytial cells with atypical enlarged pneumocytes characterized by large nuclei, amphophilic granular cytoplasm, and prominent nucleoli), which resembled the pathological changes that occurred with SARS-CoV and MERS-CoV infection. Liver histological examination showed moderate microvascular steatosis and mild lobular activity, but it is unknown whether it was caused by the virus or drug-induced liver injury. No obvious histological changes were observed in heart tissue (Xu et al., 2020). In a 72-year-old man, the histological appearance of the lung was similar to that of the above patient. The immunostaining of the lung showed that Rp3 NP, a SARS-CoV-2 protein, was prominently expressed on alveolar epithelial cells, including damaged, desquamated cells within the alveolar space, while viral protein expression was minimally detectable in blood vessels or in the interstitial areas between alveoli (Zhang et al., 2020). Sardu et al. come up with a hypothesis that the endothelium, which also express ACE2 receptors, is a key target organ of COVID-19 with the supporting of clinical and preclinical evidence (Sardu et al., 2020). Varga et al. proved the presence of viral elements in endothelial cells, and an accumulation of inflammatory cells, indicating the virus facilitating the induction of endotheliitis in several organs. They put forward the hypothesis that it was the cause of systemic impaired microcirculatory function in different vascular beds and their clinical sequelae in COVID-19 patients and suggested anti-inflammatory anti-cytokine drugs, ACE inhibitors, and statins to stabilize the endothelium (Varga et al., 2020). More pathological studies are needed to provide new insights into the pathogenesis and to help formulate better therapeutic strategies.

## Clinical Characteristics

### Clinical Presentation

According to Huang et al., among 41 admitted hospital patients in Wuhan, common symptoms at the onset of illness included fever (98%), cough (76%), and myalgia or fatigue (44%); less common symptoms included sputum production (28%), headache (8%), hemoptysis (5%), and diarrhea (3%). Dyspnea developed in 55% of patients, with a median time from illness onset of 8 days. Complications were acute respiratory distress syndrome (ARDS) (29%), RNAemia (a positive result for real-time RT-PCR in the plasma sample, 15%), acute cardiac injury (12%) and secondary infection (10%) (Huang et al., 2020). Among 99 patients in Wuhan, Chen et al. reported that patients had fever (83%), cough (82%), shortness of breath (31%), muscle aches (11%), confusion (9%), headache (8%), sore throat (5%), rhinorrhea (4%), chest pain (2%), diarrhea (2%), and nausea and vomiting (1%). Many patients presented with organ function damage, including ARDS (17%), acute respiratory injury (8%), acute renal injury (3%), septic shock (4%), and ventilator-associated pneumonia (1%) (Chen N. et al., 2020). Wang et al. reported that the most common symptoms among 138 patients at the onset of illness were fever (98.6%), fatigue (69.6%), dry cough (59.4%), myalgia (34.8%), and dyspnea (31.2%), and less common symptoms included headache (6.5%), dizziness (9.4%), abdominal pain (2.2%), diarrhea (10.1%), nausea (10.1%), and vomiting (3.6%). The median time from the onset of symptoms to dyspnea was 5.0 days, to hospital admission was 7.0 days and to ARDS was 8.0 days. Common complications were shock (8.7%), ARDS (19.6%), arrhythmia (16.7%), and acute cardiac injury (7.2%) (Wang D. et al., 2020). Chang et al. reported that patients presented with fever (92%), with a maximum temperature of 38.4°C; upper airway congestion (62%); cough (46%); myalgia (23%); and headache (23%) based on 13 cases in Beijing (Chang et al., 2020). Children even presented with Kawasaki-like disease (non-purulent conjunctivitis, polymorphic rash, mucosal changes, and swollen extremities) during the SARS-CoV-2 epidemic (Verdoni et al., 2020; Viner and Whittaker, 2020).

Based on the above studies, the clinical symptoms of COVID-19 are non-specific. Common symptoms are fever, cough, myalgia, fatigue or dyspnea, and some patients may present with sputum production, hemoptysis, diarrhea, nausea, vomiting, headache, confusion, dizziness, sore throat, rhinorrhea, or chest pain, or they may even be asymptomatic. Some patients may show rapid progression to complications such as ARDS, acute respiratory injury, RNAemia, acute cardiac injury, acute renal injury, septic shock, ventilator-associated pneumonia, or even death (Chang et al., 2020; Chen N. et al., 2020; Huang et al., 2020; Pan et al., 2020; Wang D. et al., 2020). The reason for these differences in symptoms and organ function damage might be due to the widespread distribution of ACE2 in multiple organs that provide possible routes of entry for the virus (Hamming et al., 2004).

### Laboratory Tests

Patients with COVID-19 might have leukopenia (white blood cell count <4 × 10?/L); lymphopenia (lymphocyte count <1.0 × 10?/L); abnormal platelet counts; lower levels of hemoglobin; and higher levels of hypersensitive troponin I (hs-cTnI), C-reactive protein, and plasma concentrations of IL1B, IL1RA, IL7, IL8, IL9, IL10, basic FGF, GCSF, GM-CSF, IFNγ, IP10, MCP1, MIP1A, MIP1B, PDGF, TNFα, and VEGF. Some might have liver function abnormalities, with elevated alanine aminotransferase (ALT) or aspartate aminotransferase (AST) levels, some might have abnormal myocardial zymograms with creatine kinase and lactate dehydrogenase above the normal range, and some might have renal function damage, with the elevation of blood urea nitrogen or serum creatinine. ICU patients might have higher levels of white blood cells; neutrophil counts; prothrombin time; D-dimer; creatine kinase; and plasma IL2, IL7, IL10, GCSF, IP10, MCP1, MIP1A, and TNFα (Chang et al., 2020; Chen N. et al., 2020; Huang et al., 2020; Wang D. et al., 2020). Reproductive-aged male patients were reported to have elevated serum luteinizing hormone (LH) and decreased ratio of testosterone to LH and follicle stimulating hormone to LH, indicating impaired gonadal function (Ma et al., 2020).

SARS-CoV-2 can be detected in nasopharyngeal and oropharyngeal swabs, blood, bronchoalveolar and fibrobronchoscopy brush biopsy lavage fluid, saliva, sputum, and stool specimens, with no evidence in urine (To et al., 2020; Wang W. et al., 2020). There's a lower viral load and earlier viral clearance in patients with mild COVID-19 (Horton, 2020).

### Imaging Presentation

All patients with COVID-19 might have an abnormal chest CT imaging at presentation, though some might be negative in the early stage, and all lung segments can be involved with a slight predilection for the right lower lobe. The mean number of involved lung segments was ~10. Most patients (over 75%) had bilateral lung involvement, with peripheral and diffuse distribution. The typical patterns of CT imaging were ground-glass opacity, in addition to ill-defined margins, smooth or irregular, interlobular septal thickening, air bronchogram, crazy paving pattern, and a thickening of the adjacent pleura. Less common patterns included nodules, cystic changes, bronchiolectasis, pleural effusion, and lymphadenopathy. The extent of disease on CT imaging showed rapid progress during the first 2 weeks and gradually decreased in the third week, which is consistent with the clinical course of the disease (Chen N. et al., 2020; Huang et al., 2020; Kanne, 2020; Shi et al., 2020; Wang D. et al., 2020). Fang et al. found that the sensitivity of chest CT was greater than that of RT-PCR for the diagnosis of COVID-19, and they supported the use of chest CT for COVID-19 screening in patients with clinical and epidemiological features when RT-PCR testing is negative (Fang et al., 2020).

## Diagnosis

The following diagnostic criteria were based on “the diagnosis and treatment of pneumonia with the novel coronavirus infection (trial version 6)” published by the General Office of the National Health Commission and the Office of the National Administration of Traditional Chinese Medicine.

### Suspected Case

Epidemiological history: (1) travel history or residence history in Wuhan or surrounding areas or other communities with reported cases within 14 days before onset; (2) a history of exposure to a patient with SARS-CoV-2 (nucleic acid positive) infection within 14 days before onset; (3) contact with patients who had fever or respiratory symptoms from Wuhan or surrounding areas or other communities with reported cases within 14 days before onset; and (4) a clustering of patients. Clinical manifestations: (1) fever and/or respiratory symptoms; (2) with the imaging characteristics mentioned above; and (3) the total number of white blood cells in the early stage of the disease is normal or decreased, or the lymphocyte count is reduced. Anyone who has one of the epidemiological histories and any two of the clinical manifestations or anyone has no clear epidemiological history and three of the clinical manifestations is considered to have a suspected case of COVID-19.

### Confirmed Case

(1) Positive nucleic acid of SARS-CoV-2 in respiratory tract or blood samples as determined by real-time fluorescent RT-PCR; and (2) the gene sequencing of virus in respiratory tract or blood samples is highly homologous to SARS-CoV-2. Any suspected case with one of these pathogenic indications is considered to have a confirmed case of COVID-19.

### Differential Diagnosis

Differential diagnosis mainly involves differentiation from other known viral pneumonias, such as those from the influenza virus, parainfluenza virus, adenovirus, respiratory syncytial virus, rhinovirus, human partial pneumonovirus, SARS-CoV, *Mycoplasma pneumoniae, Chlamydia pneumoniae* and bacterial pneumonia. In addition, COVID-19 needs to be differentiated from other non-infectious diseases, such as vasculitis, dermatomyositis, and organic pneumonia.

## Clinical Management

### Determine the Treatment Site According to the Condition

Patients with suspected and confirmed cases should be isolated and treated in designated hospitals with effective isolation conditions and protection conditions, patients with suspected cases should be isolated and treated in a single room, and patients with confirmed cases can be treated in the same ward by more than one person. Patients with critical cases should be admitted to the ICU as soon as possible.

### Supportive Therapy

Patients should rest in bed, ensuring adequate heat, water and electrolyte balance to maintain internal environment stability, being closely monitored for vital signs and oxygen saturation. Effective oxygen therapy should be provided in a timely manner. If the patient has no evidence of shock, conservative infusion therapy is recommended. Gamma globulins can be used as appropriate in patients with severe cases (Jin Y. H. et al., 2020).

### Antiviral Treatment

There is currently no evidence-based specific drug treatment against COVID-19. α-Interferon atomization inhalation, oral lopinavir/ritonavir, ribavirin, and chloroquine phosphate can be considered (Jin Y. H. et al., 2020). Wang et al. found that remdesivir, a promising antiviral drug (including SARS/MERS-CoV5), and chloroquine, an antimalarial and autoimmune disease drug, effectively inhibited SARS-CoV-2 infection *in vitro* (Wang M. et al., 2020). However, in a retrospective analysis in the US, Magagnoli et al. found that there's no evidence of hydroxychloroquine reduced the risk of mechanical ventilation while increased overall mortality was observed in patients treated with hydroxychloroquine alone (Magagnoli et al., 2020). In a multicentre, open label, randomized controlled trial, hydroxychloroquine did not result in a significantly higher probability of negative conversion while adverse events were higher in hydroxychloroquine recipients (Tang et al., 2020). Several other clinical trials have also found no evidence to support the use of hydroxychloroquine in patients with covid-19 (Geleris et al., 2020; Mahevas et al., 2020; Rosenberg et al., 2020). Evidence that intravenous remdesivir was administered in the first case of COVID-19 in the United States and the patient's condition improved later suggested that remdesivir is worthy of clinical trials (Holshue et al., 2020). Nguyen et al. proposed a CRISPR/Cas13d system as a potential therapy that contained guide RNAs to specifically target the virus RNA genome and Cas13d effector using adeno-associated virus (AAV) as a vehicle to digest the viral RNA genome without affecting the human transcriptome (Nguyen T. M. et al., 2020). Richardson et al. suggested that an AAK1-binding drug, baricitinib, could be trialed because it might interrupt receptor-mediated endocytosis and intracellular assembly of viral particles (Richardson et al., 2020). And Stebbing et al. supported baricitinib may be of use in countering COVID-19 because its high affinity for AAK1, once-daily oral dosing and acceptable side-effect. They also suggested the combination of baricitinib with the directacting antivirals (Stebbing et al., 2020). It was recommended in a Comment in The Lancet Infect Dis to test the convalescent plasma transfusion in SARS-CoV-2-infected patients (Chen L. et al., 2020), and there are many clinical trials going on with this approach (Table 2). In spite of this, Casadevall and Pirofski discussed the potential risks of this approach including known risks associated with transfer of blood substances and theoretical risks of the phenomenon of antibody-dependent enhancement of infection and attenuation of immune response to the virus (Casadevall and Pirofski, 2020). This is an important issue, as SARS-CoV-2 is reported to be present in some blood samples by Wang W. et al. (2020), and additionally, this virus is known to have different subtypes (Morais Júnior et al., 2020), which in turn, may raise the concern of infecting the patients with a different subtype. Additionally, Anti–spike IgG is reported to induce severe acute lung injury by SARS-CoV, a close relative of this virus (Liu et al., 2019).

**Table 2:** Representative ongoing clinical trials of antiviral therapy for COVID-19.

**Registration number**	**Drug**	**Phase**
ChiCTR2000030718/ChiCTR2000030054/ChiCTR2000029992/ChiCTR2000029988	Chloroquine phosphate	IV
ChiCTR2000030704	Bufonis venenum	IV
ChiCTR2000030702/ ChiCTR2000030627/ChiCTR2000030381/ChiCTR2000030312/ChiCTR2000030046/ChiCTR2000029975	Convalescent plasma	0
ChiCTR2000030701	Prolongin	0
ChiCTR2000030545/ChiCTR2000029954	Honeysuckle oral liquid	IV
ChiCTR2000030535	Ebastine	IV
ChiCTR2000030509	NK Cells	0
ChiCTR2000030487/ChiCTR2000030424/ChiCTR2000030041/ChiCTR2000029853	Azvudine	0
ChiCTR2000030480/ChiCTR2000030013	Human interferon α1b	IV
ChiCTR2000030475	Cytosorb	0
ChiCTR2000030442	Combination of tocilizumab, IVIG and CRRT	0
ChiCTR2000030398	Bismuth potassium citrate	N/A
ChiCTR2000030333	Pirfenidone	0
ChiCTR2000030254/ChiCTR2000030113/ChiCTR2000029996	Farpiravir	0
ChiCTR2000030218	Combination of pinavir and ritonavir	N/A
ChiCTR2000030170	Jakotinib hydrochloride	0
ChiCTR2000030167	Recombinant human interleukin-2	0
ChiCTR2000030138/ChiCTR2000030088/ChiCTR2000029990	Human mesenchymal stem cells	II
ChiCTR2000030089	Adalimumab	IV
ChiCTR2000030001	Triazavirin	III
ChiCTR2000029898/ChiCTR2000029868/ChiCTR2000029761	Hydroxychloroquine sulfate	IV
ChiCTR2000029741	Chloroquine and lopinavir/ Ritonavir	IV
NCT04287686	Recombinant human angiotensin-converting enzyme 2	N/A
NCT04292899/NCT04292730/NCT04252664	Remdesivir	III
NCT04305106/NCT04275414	Bevacizumab	N/A
NCT04273529/NCT04273581	Thalidomide	II
NCT04280588	Fingolimod	II
NCT04261426	Intravenous Immunoglobulin	II and III
NCT04306393/NCT04290871	Nitric Oxide	II
NCT04252274	Darunavir and obicistat	III

Recently, a SARS-CoV-2 protein interaction map was addressed and 66 druggable human proteins or host factors was identified. Further screening in multiple viral assays identified two groups of drugs showing antiviral activity including inhibitors of mRNA translation and predicted regulators of the Sigma1 and Sigma2 receptors (Table 3) (Gordon et al., 2020). Bojkova et al. identified the SARS-CoV-2 infection profile through translatome and proteome proteomics which revealed cellular pathway reshaped such as translation, splicing, carbon metabolism and nucleic acid metabolism. Drugs inhibiting these pathways prevent the virus from replicating inside the cells (Table 3) (Bojkova et al., 2020).

**Table 3:** List of different predicted possible drugs.

**Drug types**	**Drugs**
Protein biogenesis inhibitors	Zotatifin Ternatin-4 PS3061
Ligands of the Sigma1 and Sigma2 receptors	Haloperidol PB28 PD-144418 Hydroxychloroquine
SigmaR1/R2 active drugs clemastine	Cloperastine progesterone
Compounds interfering with nucleic acid metabolism	Ribavirin
Proteostasis perturbation	NMS-873

There are many ongoing antiviral therapy clinical trials for COVID-19 (Table 2); however, the preliminary results of these trials are questionable. The sample size of some trials is obviously insufficient, many clinical trial designs do not adhere to the principles of randomization and control, and many do not use blind evaluation. Some antiviral therapies, similar to drugs that fight the cold, flu and others, are even not worth trying.

### Corticosteroid Therapy

Corticosteroids are not recommended for use in patients with SARS-CoV-2-induced lung injury or shock (Russell et al., 2020). Using corticosteroids should follow these basic principles: (1) the benefits and harms of corticosteroids should be carefully weighed before their use; (2) corticosteroids should be used prudently in critically ill patients with COVID-19; (3) for patients with hypoxemia due to underlying diseases or who regularly use corticosteroids for chronic diseases, further use of corticosteroids should be cautious; and (4) the dosage of corticosteroids should be low to moderate (≤0.5–1 mg/kg per day methylprednisolone or equivalent) and the duration of use should be short (≤7 days) (Shang L. et al., 2020; Zhao et al., 2020). Pathological findings of pulmonary edema and hyaline membrane formation indicate that appropriate use of corticosteroids and ventilator support should be considered for severe patients (Xu et al., 2020). Evidence has shown that treatment with methylprednisolone is also associated with better outcomes among patients who develop ARDS (Wu C. et al., 2020).

### Treatment of Patients With Severe and Critical Cases

The principle of the treatment of patients with severe and critical cases is that on the basis of symptomatic treatment, we should actively prevent and treat complications, treat basic diseases, prevent secondary infection, and support organ function in a timely manner. The patient should be transferred to invasive mechanical ventilation in a timely manner if the patient's condition does not improve after 2 h of non-invasive mechanical ventilation or if the patient is unable to tolerate non-invasive ventilation with increased airway secretions, severe cough, or unstable hemodynamics. If necessary, prone position ventilation, lung retraction, or extracorporeal membrane oxygenation (ECMO) should be adopted. On the basis of full fluid resuscitation, microcirculation should be improved, vasoactive drugs should be used, and hemodynamic monitoring should be performed if necessary (Jin Y. H. et al., 2020; Working Group of 2019 Novel Coronavirus, 2020).

### Pregnant Women With COVID-19

For pregnant women with suspected infection, SARS-CoV-2 nucleic acid testing should be performed. Asymptomatic confirmed pregnant women should self-monitor for clinical features of COVID-19 for at least 14 days at home. Patients who have recovered from mild symptoms should be monitored bimonthly with fetal growth ultrasounds and Doppler ultrasounds. Otherwise, patients should be managed by a multidisciplinary team. Delivery timing depends on the week of gestation and maternal, fetal, and delivery conditions, and vaginal delivery with eventual instrumental delivery is favored. Emergency cesarean delivery should be managed in conditions of septic shock, acute organ failure, and fetal distress, or termination of the pregnancy if legal before fetal viability (Favre et al., 2020; Rasmussen et al., 2020).

### Neonates With COVID-19

Neonates with COVID-19 should be isolated and clinically monitored, but neonatal intensive care unit (NICU) admission is not necessary except in the case of life-threatening situations. Patients should be managed by a multidisciplinary team. Respiratory support policies should follow international guidelines. Antiviral drugs (remdesivir or lopinavir/ritonavir) can be considered compassionate treatment after careful consideration of the risk/benefit ratio and technical issues. Antibiotics, especially broad-spectrum antibiotics, are not allowed unless there is secondary bacterial infection (De Luca, 2020; Wang L. et al., 2020).

### Removal From Isolation and Discharge Standards

Patients meeting the following conditions can be discharged from isolation: (1) body temperature has returned to normal for more than 3 days; (2) respiratory symptoms have been improved significantly; (3) obvious reduction in inflammation in lung imaging; and (4) two consecutive negative respiratory pathogen nucleic acid tests (sampling time interval of at least 1 day).

## Prognosis

Though SARS-CoV-2 is highly infectious, most of the patients have mild manifestations (80.9%) and a low overall case-fatality rate (2.3%). Critical cases account for 4.7%, and the crude case-fatality rate is 49% (Epidemiology Working Group for NCIP Epidemic Response Chinese Center for Disease Control Prevention, 2020). As of March 5, 2020, a report showed case-fatality risk in four populations of China (3.5%); China, excluding Hubei Province (0.8%); 82 countries, territories, and areas (4.2%); and on a cruise ship (0.6%) (Wilson et al., 2020). Resently, Baud et al. reported that mortality rates was 5.6% for China and 15.2% outside of China (Baud et al., 2020). According to the results updated on May 18th, Belgium has relatively high case fatality rates (16.34%), followed by France (15.65%), UK (14.21), Italy (14.15), Hungary (13.07), Netherlands (12.91%), Sweden (12.21%) and USA (5.95), China (5.59) so on (Oke). Old age; male sex; a history of smoking; higher Sequential Organ Failure Assessment (SOFA) score; maximum body temperature on admission; underlying diseases such as hypertension, chronic respiratory disease, cardiovascular disease, diabetes and cancer; respiratory failure; albumin; C-reactive protein; and progressive radiographic deterioration on follow-up CT (pleural effusion, lymphadenopathy) might be risk factors for poor prognosis in patients with COVID-19 pneumonia (Applegate and Ouslander, 2020; Epidemiology Working Group for NCIP Epidemic Response Chinese Center for Disease Control Prevention, 2020; Liu et al., 2020a; Shi et al., 2020; Zhou F. et al., 2020). High fever was associated with ARDS development, but it was also associated with better outcomes among patients with ARDS (Wu C. et al., 2020). It is interesting to note that genetic variability may affect susceptibility to and severity of COVID-19, since Nguyen et al. found that HLA-B^*^46:01 had the fewest predicted binding peptides for SARS-CoV-2, indicating people with this allele may be particularly vulnerable to COVID-19 while HLA-B^*^15:03 was converse (Nguyen A. et al., 2020).

## Infection Control and Prevention

There is an urgent need for infection control and prevention in the face of such a severe epidemic. (1) Air disinfection using disinfectants and alcohol has no value and should be avoided. (2) Public health education based on scientific evidence needs to be timely and objectively avoid confusion and chaos, and the spread of fake news and misinformation should be forbidden. (3) Animal source or sources should be identified, and transmission amplification events should be prevented. (4) The wearing of masks would probably intercept the transmission of the virus in close person-to-person contacts, though WHO recommends against it in community settings because of a lack of evidence. (5) Services to amplify the ability to absorb and adapt to shock should be integrated. (6) Diagnostics, therapeutics and vaccines are urgently needed. (7) Attention should be paid to the physical and mental impacts on children and adolescents caused by home confinement. The government, communities, schools, and parents should work together to minimize the impact. (8) Loneliness and anger can occur in quarantined people, and attention should be paid to their mental health care (Legido-Quigley et al., 2020; Leung et al., 2020; Wang G. et al., 2020; Xiao and Torok, 2020; Zandifar and Badrfam, 2020).

## Discussion

The outbreak of COVID-19 poses a serious clinical threat to the general population worldwide. With the scientists' efforts, we are gradually understanding different aspects of COVID-19, but knowledge about this disease is still limited with unresolved issues such as tracing the index case, the development of vaccine and antiviral drugs, the mutation rate of this RNA virus, and the sequelae induced by COVID-19.

Different countries have different responses to the outbreak. In china, many cities were closed and social contacts were limited. Close contact tracing management was carried out to detect and effectively control the source of infection at an early stage. Large-scale activities were canceled and the resumption of work in factories and the opening of the school were delayed. People were encouraged to wear masks and pay attention to hand hygiene (Chen S. et al., 2020; Chen W. et al., 2020). In Italy, measures like interruptions of air traffic from China and quarantines for Italian travelers in China were taken to restrict viral spread. An emergency task force of Lombardy and the authorities of local health were established (Grasselli et al., 2020; Spina et al., 2020). In the United States, the president signed a “Proclamation on Suspension of Entry as Immigrants and Non-immigrants of Persons who Pose a Risk of Transmitting 2019 Novel Coronavirus” (Patel and Jernigan, 2020). However, there's basically no quarantine, not to mention no closure of the city. Singapore has steadily built up its outbreak preparedness since the 2003 SARS outbreak and a Multi–Ministry Task Force was set up to handl of the crisis. They aimed to identify as many cases as possible and all suspected and confirmed cases were immediately isolated in hospital. The patients were managed by a network of preparedness facilities. Besides, prevention of imported cases by temperature and health screening were carried out (Lee et al., 2020).

To our surprise, the UK government's strategy for fighting COVID-19 is a markedly different approach, which is to push for “herd immunity” to the virus by allowing at least 40 million Britons to become infected in the hopes of building up a long-term, society-wide resistance to the disease. This approach is opposed by multiple scientists. In our opinion, this strategy is ridiculous. Herd immunity is defined as the resistance of a group to attack by a disease to which a large proportion of the members are immune, thus lessening the likelihood of a patient with a disease coming into contact with a susceptible individual (Fox et al., 1971). The main way of obtaining herd immunity is vaccination (Anderson and May, 1985), while the UK government's policy is going to sacrifice countless people, which we think is inhumane for a civilized society. In addition, viruses can mutate, and there is no evidence that the immunity of the cured is permanent. Therefore, we are strongly against this policy. At present, the vast majority of European and American countries still do not adopt China's strategy of “collect as much as possible,” but let a large number of mild patients isolated at home, which increases the risk of transmission. Karin et al. proposed a cyclic schedule of 4-day work and 10-day lockdown by mathematical models which could can prevent resurgence of the epidemic while providing part-time employment. It provides a good way for the government to manage the epidemic (Karin et al., 2020).

The top priorities are isolation, and vaccine and antiviral drug development. We believe with the effort of the whole world and through lessons learned from the MERS and SARS outbreaks, the final victory is not far away.

## Author Contributions

XF and SG supervised the project and provided direction and guidance throughout the preparation of this manuscript. PC collected and prepared the related papers. JY wrote the manuscript. All authors read and approved the final manuscript.

## Conflict of Interest

The authors declare that the research was conducted in the absence of any commercial or financial relationships that could be construed as a potential conflict of interest.

## Abbreviations

COVID-19: Corona Virus Disease 2019
SARS-CoV-2: SARS-coronavirus 2
PHEIC: public health emergency of international concern
WHO: World Health Organization
CSG: Coronavirus Study Group
CoVs: Coronaviruses
SARS: severe acute respiratory syndrome
R_0_: reproduction number
hs-cTnI: hypersensitive troponin I
ALT: alanine aminotransferase
AST: aspartate aminotransferase
AAV: adeno-associated virus
NICU: neonatal intensive care units.

## References

[B1] Andersen K. G. Rambaut A. Lipkin W. I. Holmes E. C. Garry R. F. (2020). The proximal origin of SARS-CoV-2. Nat. Med. 26, 450–452. 10.1038/s41591-020-0820-9 32284615 PMC7095063

[B2] Anderson R. M. May R. M. (1985). Vaccination and herd immunity to infectious diseases. Nature 318, 323–329. 10.1038/318323a0 3906406

[B3] Applegate W. B. Ouslander J. G. (2020). COVID-19 presents high risk to older persons. J. Am. Geriatr. Soc. 68:681. 10.1111/jgs.16426 32154911 PMC7166412

[B4] Backer J. A. Klinkenberg D. Wallinga J. (2020). Incubation period of 2019 novel coronavirus (2019-nCoV) infections among travellers from Wuhan, China, 20-28 January 2020. Eur. Surveill. 25:2000062. 10.2807/1560-7917.ES.2020.25.5.2000062 32046819 PMC7014672

[B5] Baud D. Qi X. Nielsen-Saines K. Musso D. Pomar L. Favre G. (2020). Real estimates of mortality following COVID-19 infection. Lancet Infect. Dis. 10.1016/S1473-3099(20)30195-X 32171390 PMC7118515

[B6] Bojkova D. Klann K. Koch B. Widera M. Krause D. Ciesek S.. (2020). Proteomics of SARS-CoV-2-infected host cells reveals therapy targets. Nature. 10.1038/s41586-020-2332-7 32408336 PMC7616921

[B7] Cai J. Sun W. Huang J. Gamber M. Wu J. He G. (2020). Indirect virus transmission in Cluster of COVID-19 Cases, Wenzhou, China, 2020. Emerg. Infect. Dis. 26, 1343–1345. 10.3201/eid2606.200412 32163030 PMC7258486

[B8] Casadevall A. Pirofski L. A. (2020). The convalescent sera option for containing COVID-19. J. Clin. Invest. 130, 1545–1548. 10.1172/JCI138003 32167489 PMC7108922

[B9] Chai P. Yu J. Ge S. Jia R. Fan X. (2020). Genetic alteration, RNA expression, and DNA methylation profiling of coronavirus disease 2019 (COVID-19) receptor ACE2 in malignancies: a pan-cancer analysis. J. Hematol. Oncol. 13:43. 10.1186/s13045-020-00883-5 32366279 PMC7197362

[B10] Chang L. M. Wei L. Xie L. Zhu G. Dela Cruz C.S.. (2020). Epidemiologic and Clinical characteristics of novel coronavirus infections involving 13 patients outside Wuhan, China. JAMA 323, 1092–1093. 10.1001/jama.2020.1623 32031568 PMC7042871

[B11] Chan-Yeung M. Xu R. H. (2003). SARS: epidemiology. Respirology 8, S9–S14. 10.1046/j.1440-1843.2003.00518.x 15018127 PMC7169193

[B12] Chen H. Guo J. Wang C. Luo F. Yu X. Zhang W.. (2020). Clinical characteristics and intrauterine vertical transmission potential of COVID-19 infection in nine pregnant women: a retrospective review of medical records. Lancet 395, 809–815. 10.1016/S0140-6736(20)30360-3 32151335 PMC7159281

[B13] Chen L. Xiong J. Bao L. Shi Y. (2020). Convalescent plasma as a potential therapy for COVID-19. Lancet Infect. Dis. 20, 398–400. 10.1016/S1473-3099(20)30141-9 32113510 PMC7128218

[B14] Chen N. Zhou M. Dong X. Qu J. Gong F. Han Y.. (2020). Epidemiological and clinical characteristics of 99 cases of 2019 novel coronavirus pneumonia in Wuhan, China: a descriptive study. Lancet 395, 507–513. 10.1016/S0140-6736(20)30211-7 32007143 PMC7135076

[B15] Chen S. Yang J. Yang W. Wang C. Barnighausen T. (2020). COVID-19 control in China during mass population movements at New Year. Lancet 395, 764–766. 10.1016/S0140-6736(20)30421-9 32105609 PMC7159085

[B16] Chen W. Wang Q. Li Y. Q. Yu H. L. Xia Y. Y. Zhang M. L.. (2020). [Early containment strategies and core measures for prevention and control of novel coronavirus pneumonia in China]. Zhonghua Yu Fang Yi Xue Za Zhi 54, 239–244. 10.3760/cma.j.issn.0253-9624.2020.03.003 32064856

[B17] Chen Y. Liu Q. Guo D. (2020). Emerging coronaviruses: genome structure, replication, and pathogenesis. J. Med. Virol. 92, 418–423. 10.1002/jmv.25681 31967327 PMC7167049

[B18] Crokidakis N. (2020). Data analysis and modeling of the evolution of COVID-19 in Brazil. arXiv.

[B19] Dai W. Zhang B. Su H. Li J. Zhao Y. Xie X.. (2020). Structure-based design of antiviral drug candidates targeting the SARS-CoV-2 main protease. Science abb4489. 10.1126/science.abb4489 32321856 PMC7179937

[B20] De Luca D. (2020). Managing neonates with respiratory failure due to SARS-CoV-2. Lancet Child Adolesc. Health. 4:e8. 10.1016/S2352-4642(20)30073-0 32151320 PMC7128679

[B21] Ensser A. Überla P. Überla K. (2020). Modest effects of contact reduction measures on the reproduction number of SARS-CoV-2 in the most affected European countries and the US. medRxiv. 10.1101/2020.04.20.20067538

[B22] Ensser A. Ueberla K. (2020). Determination of daily reproduction numbers of SARS-CoV2 based on death cases suggests more rapid initial spread in Italy and the United States. medRxiv. 10.1101/2020.03.28.20046094

[B23] Epidemiology Working Group for NCIP Epidemic Response and Chinese Center for Disease Control and Prevention (2020). [The epidemiological characteristics of an outbreak of 2019 novel coronavirus diseases (COVID-19) in China]. Zhonghua Liu Xing Bing Xue Za Zhi 41, 145–151. 10.3760/cma.j.issn.0254-6450.2020.02.003 32064853

[B24] Fang Y. Zhang H. Xie J. Lin M. Ying L. Pang P.. (2020). Sensitivity of chest CT for COVID-19: comparison to RT-PCR. Radiology 200432. 10.1148/radiol.2020200432 32073353 PMC7233365

[B25] Favre G. Pomar L. Qi X. Nielsen-Saines K. Musso D. Baud D. (2020). Guidelines for pregnant women with suspected SARS-CoV-2 infection. Lancet Infect. Dis. 10.1016/S1473-3099(20)30157-2 32142639 PMC7134390

[B26] Fox J. P. Elveback L. Scott W. Gatewood L. Ackerman E. (1971). Herd immunity: basic concept and relevance to public health immunization practices. Am. J. Epidemiol. 94, 179–189. 10.1093/oxfordjournals.aje.a121310 5093648

[B27] Gao Y. Yan L. Huang Y. Liu F. Zhao Y. Cao L.. (2020). Structure of the RNA-dependent RNA polymerase from COVID-19 virus. Science 368, 779–782. 10.1126/science.abb7498 32277040 PMC7164392

[B28] Geleris J. Sun Y. Platt J. Zucker J. Baldwin M. Hripcsak G.. (2020). Observational study of hydroxychloroquine in hospitalized patients with Covid-19. N. Engl. J. Med. 10.1056/NEJMoa2012410 32379955 PMC7224609

[B29] Gorbalenya A. E. Baker S. C. Baric R. S. de Groot R. J. Drosten C. Gulyaeva A. A. (2020). Severe acute respiratory syndrome-related coronavirus: the species and its viruses – a statement of the Coronavirus Study Group. bioRxiv 10.1101/2020.02.07.937862

[B30] Gordon D. E. Jang G. M. Bouhaddou M. Xu J. Obernier K. White K. M.. (2020). A SARS-CoV-2 protein interaction map reveals targets for drug repurposing. Nature. 10.1038/s41586-020-2286-9. [Epub ahead of print]. 32353859 PMC7431030

[B31] Grasselli G. Pesenti A. Cecconi M. (2020). Critical care utilization for the COVID-19 outbreak in lombardy, italy: early experience and forecast during an emergency response. JAMA 323, 1545–1546. 10.1001/jama.2020.4031 32167538

[B32] Grifoni A. Sidney J. Zhang Y. Scheuermann R. H. Peters B. Sette A. (2020). A sequence homology and bioinformatic approach can predict candidate targets for immune responses to SARS-CoV-2. Cell Host Microbe. 27, 671–80.e672. 10.1016/j.chom.2020.03.002 32183941 PMC7142693

[B33] Hamming I. Timens W. Bulthuis M. L. Lely A. T. Navis G. van Goor H. (2004). Tissue distribution of ACE2 protein, the functional receptor for SARS coronavirus. a first step in understanding SARS pathogenesis. J. Pathol. 203, 631–637. 10.1002/path.1570 15141377 PMC7167720

[B34] Hoffmann M. Kleine-Weber H. Schroeder S. Krüger N. Herrler T. Erichsen S.. (2020). SARS-CoV-2 cell entry depends on ACE2 and TMPRSS2 and is blocked by a clinically proven protease inhibitor. Cell 181, 271–80.e278. 10.1016/j.cell.2020.02.052 32142651 PMC7102627

[B35] Holshue M. L. DeBolt C. Lindquist S. Lofy K. H. Wiesman J. Bruce H. (2020). First case of 2019 novel Coronavirus in the United States. N. Engl. J. Med. 382, 929–936. 10.1056/NEJMoa2001191 32004427 PMC7092802

[B36] Horton R. (2020). Offline: COVID-19—a reckoning. Lancet 395:935. 10.1016/S0140-6736(20)30669-3 32199478 PMC7156226

[B37] Huang C. Wang Y. Li X. Ren L. Zhao J. Hu Y.. (2020). Clinical features of patients infected with 2019 novel coronavirus in Wuhan, China. Lancet 395, 497–506. 10.1016/S0140-6736(20)30183-5 31986264 PMC7159299

[B38] Jiang S. Shi Z. Shu Y. Song J. Gao G. F. Tan W.. (2020). A distinct name is needed for the new coronavirus. Lancet 395:949. 10.1016/S0140-6736(20)30419-0 32087125 PMC7124603

[B39] Jin Y. H. Cai L. Cheng Z. S. Cheng H. Deng T. Fan Y. P.. (2020). A rapid advice guideline for the diagnosis and treatment of 2019 novel coronavirus (2019-nCoV) infected pneumonia (standard version). Mil. Med. Res. 7:4. 10.1186/s40779-020-0233-6 32029004 PMC7003341

[B40] Jin Z. Du X. Xu Y. Deng Y. Liu M. Zhao Y.. (2020). Structure of M(pro) from SARS-CoV-2 and discovery of its inhibitors. Nature 10.1038/s41586-020-2223-y 32272481

[B41] Kanne J. P. (2020). Chest CT findings in 2019 novel Coronavirus (2019-nCoV) infections from Wuhan, China: key points for the radiologist. Radiology 295, 16–17. 10.1148/radiol.2020200241 32017662 PMC7233362

[B42] Karin O. Bar-On Y. M. Milo T. Katzir I. Mayo A. Korem Y. (2020). Adaptive cyclic exit strategies from lockdown to suppress COVID-19 and allow economic activity. medRxiv 10.1101/2020.04.04.20053579

[B43] Lam T. T. Y. Shum M. H.H. Zhu H.C. Tong Y.G. Ni X.B. Liao Y.S.. (2020). Identification of 2019-nCoV related coronaviruses in Malayan pangolins in southern China. bioRxiv 10.1101/2020.02.13.945485 32218527

[B44] Lan J. Ge J. Yu J. Shan S. Zhou H. Fan S.. (2020). Structure of the SARS-CoV-2 spike receptor-binding domain bound to the ACE2 receptor. Nature 581, 215–220. 10.1038/s41586-020-2180-5 32225176

[B45] Lauer S. A. Grantz K. H. Bi Q. Jones F. K. Zheng Q. Meredith H. R.. (2020). The incubation period of coronavirus disease 2019 (COVID-19) from publicly reported confirmed cases: estimation and application. Ann. Intern. Med. 172, 577–582. 10.7326/M20-0504 32150748 PMC7081172

[B46] Lee V. J. Chiew C. J. Khong W. X. (2020). Interrupting transmission of COVID-19: lessons from containment efforts in Singapore. J. Travel Med. 27:taaa039. 10.1093/jtm/taaa039 32167146 PMC7107552

[B47] Legido-Quigley H. Asgari N. Teo Y. Y. Leung G. M. Oshitani H. Fukuda K.. (2020). Are high-performing health systems resilient against the COVID-19 epidemic? Lancet 395, 848–850. 10.1016/S0140-6736(20)30551-1 32151326 PMC7124523

[B48] Leung C. C. Lam T. H. Cheng K. K. (2020). Mass masking in the COVID-19 epidemic: people need guidance. Lancet 395:945. 10.1016/S0140-6736(20)30520-1 32142626 PMC7133583

[B49] Li Q. Guan X. Wu P. Wang X. Zhou L. Tong Y.. (2020). Early transmission dynamics in Wuhan, China, of novel coronavirus-infected pneumonia. N. Engl. J. Med. 382, 1199–1207. 10.1056/NEJMoa2001316 31995857 PMC7121484

[B50] Li Y. Zhao R. Zheng S. Chen X. Wang J. Sheng X.. (2020). Lack of vertical transmission of severe acute respiratory syndrome Coronavirus 2, China. Emerg. Infect. Dis. 26, 1335–1336. 10.3201/eid2606.200287 32134381 PMC7258467

[B51] Linton N. M. Kobayashi T. Yang Y. Hayashi K. Akhmetzhanov A. R. Jung S. M.. (2020). Incubation period and other epidemiological characteristics of 2019 novel coronavirus infections with right truncation: a statistical analysis of publicly available case data. J. Clin. Med. 9:538. 10.3390/jcm9020538 32079150 PMC7074197

[B52] Liu L. Wei Q. Lin Q. Fang J. Wang H. Kwok H.. (2019). Anti-spike IgG causes severe acute lung injury by skewing macrophage responses during acute SARS-CoV infection. JCI Insight. 4:e123158. 10.1172/jci.insight.123158 30830861 PMC6478436

[B53] Liu W. Tao Z. W. Lei W. Ming-Li Y. Kui L. Ling Z. (2020a). Analysis of factors associated with disease outcomes in hospitalized patients with 2019 novel coronavirus disease. Chin. Med. J. 10.1097/CM9.0000000000000775 PMC7147279 32118640

[B54] Liu W. Zhang Q. Chen J. Xiang R. Song H. Shu S.. (2020b). Detection of Covid-19 in children in early january 2020 in Wuhan, China. N. Engl. J. Med. 382, 1370–1371. 10.1056/NEJMc2003717 32163697 PMC7121643

[B55] Lowe B. Bopp B. (2020). COVID-19 vaginal delivery – a case report. Aust. Nz. J. Obstet. Gyn. 10.1111/ajo.13173 32294229 PMC7262173

[B56] Lu C. W. Liu X. F. Jia Z. F. (2020). 2019-nCoV transmission through the ocular surface must not be ignored. Lancet 395:e39. 10.1016/S0140-6736(20)30313-5 32035510 PMC7133551

[B57] Lu R. Zhao X. Li J. Niu P. Yang B. Wu H.. (2020). Genomic characterisation and epidemiology of 2019 novel coronavirus: implications for virus origins and receptor binding. Lancet 395, 565–574. 10.1016/S0140-6736(20)30251-8 32007145 PMC7159086

[B58] Ma L. Xie W. Li D. Shi L. Mao Y. Xiong Y. (2020). Effect of SARS-CoV-2 infection upon male gonadal function: a single center-based study. medRxiv 10.1101/2020.03.21.20037267

[B59] Magagnoli J. Narendran S. Pereira F. Cummings T. Hardin J. W. Sutton S. S. (2020). Outcomes of hydroxychloroquine usage in United States veterans hospitalized with Covid-19. medRxiv 10.1101/2020.04.16.20065920 PMC7274588 32838355

[B60] Mahevas M. Tran V. T. Roumier M. Chabrol A. Paule R. Guillaud C.. (2020). Clinical efficacy of hydroxychloroquine in patients with covid-19 pneumonia who require oxygen: observational comparative study using routine care data. BMJ 369:m1844. 10.1136/bmj.m1844 32409486 PMC7221472

[B61] Memish Z. A. Perlman S. Van Kerkhove M. D. Zumla A. (2020). Middle East respiratory syndrome. Lancet 395, 1063–1077. 10.1016/S0140-6736(19)33221-0 32145185 PMC7155742

[B62] Morais Júnior I. J. Polveiro R. C. Souza G. M. Bortolin D. I. Sassaki F. T. Lima A. T. M. (2020). The global population of SARS-CoV-2 is composed of six major subtypes. bioRxiv 10.1101/2020.04.14.040782 PMC7588421 33106569

[B63] Nguyen A. David J. K. Maden S. K. Wood M. A. Weeder B. R. Nellore A.. (2020). Human leukocyte antigen susceptibility map for SARS-CoV-2. medRxiv 10.1101/2020.03.22.20040600 32303592 PMC7307149

[B64] Nguyen T. M. Zhang Y. Pandolfi P. P. (2020). Virus against virus: a potential treatment for 2019-nCov (SARS-CoV-2) and other RNA viruses. Cell Res. 30, 189–190. 10.1038/s41422-020-0290-0 32071427 PMC7054296

[B65] Oke J. H. C. (2020). Global COVID-19 Case Fatality Rates. CEBM Research. Available online at: https://www.cebm.net/global-covid-19-case-fatality-rates (accessed May 18, 2020).

[B66] Pan X. Chen D. Xia Y. Wu X. Li T. Ou X.. (2020). Asymptomatic cases in a family cluster with SARS-CoV-2 infection. Lancet Infect. Dis. 20, 410–411. 10.1016/S1473-3099(20)30114-6 32087116 PMC7158985

[B67] Patel A. Jernigan D. B. (2020). Initial public health response and interim clinical guidance for the 2019 novel coronavirus outbreak - United States, December 31, 2019-February 4, 2020. Morb. Mortal. Wkly. Rep. 69, 140–146. 10.15585/mmwr.mm6905e1 32027631 PMC7004396

[B68] Rasmussen S. A. Smulian J. C. Lednicky J. A. Wen T. S. Jamieson D. J. (2020). Coronavirus Disease 2019 (COVID-19) and pregnancy: what obstetricians need to know. Am. J. Obstet. Gynecol. 222, 415–426. 10.1016/j.ajog.2020.02.017 32105680 PMC7093856

[B69] Richardson P. Griffin I. Tucker C. Smith D. Oechsle O. Phelan A.. (2020). Baricitinib as potential treatment for 2019-nCoV acute respiratory disease. Lancet 395, e30–e31. 10.1016/S0140-6736(20)30304-4 32032529 PMC7137985

[B70] Rosenberg E. S. Dufort E. M. Udo T. Wilberschied L. A. Kumar J. Tesoriero J.. (2020). Association of treatment with hydroxychloroquine or azithromycin with in-hospital mortality in patients with COVID-19 in New York state. JAMA 11:e208630. 10.1001/jama.2020.8630 32392282 PMC7215635

[B71] Rothe C. Schunk M. Sothmann P. Bretzel G. Froeschl G. Wallrauch C.. (2020). Transmission of 2019-nCoV infection from an asymptomatic contact in Germany. N. Engl. J. Med. 382, 970–971. 10.1056/NEJMc2001468 32003551 PMC7120970

[B72] Russell C. D. Millar J. E. Baillie J. K. (2020). Clinical evidence does not support corticosteroid treatment for 2019-nCoV lung injury. Lancet 395, 473–475. 10.1016/S0140-6736(20)30317-2 32043983 PMC7134694

[B73] Sardu C. Gambardella J. Morelli M. Wang X. Marfella R. Santulli G. (2020). Is COVID-19 an endothelial disease? clinical and basic evidence. Preprints 2020:2020040204. 10.20944/preprints202004.0204.v1 32403217 PMC7290769

[B74] Shang J. Ye G. Shi K. Wan Y. Luo C. Aihara H.. (2020). Structural basis of receptor recognition by SARS-CoV-2. Nature 581, 221–224. 10.1038/s41586-020-2179-y 32225175 PMC7328981

[B75] Shang L. Zhao J. Hu Y. Du R. Cao B. (2020). On the use of corticosteroids for 2019-nCoV pneumonia. Lancet 395, 683–684. 10.1016/S0140-6736(20)30361-5 32122468 PMC7159292

[B76] Shi H. Han X. Jiang N. Cao Y. Alwalid O. Gu J.. (2020). Radiological findings from 81 patients with COVID-19 pneumonia in Wuhan, China: a descriptive study. Lancet Infect. Dis. 20. 10.1016/S1473-3099(20)30086-4 32105637 PMC7159053

[B77] Spina S. Marrazzo F. Migliari M. Stucchi R. Sforza A. Fumagalli R. (2020). The response of Milan's emergency medical system to the COVID-19 outbreak in Italy. Lancet 395, e49–e50. 10.1016/S0140-6736(20)30493-1 32119824 PMC7124430

[B78] Stebbing J. Phelan A. Griffin I. Tucker C. Oechsle O. Smith D.. (2020). COVID-19: combining antiviral and anti-inflammatory treatments. Lancet. Infect. Dis. 20, 400–402. 10.1016/S1473-3099(20)30132-8 32113509 PMC7158903

[B79] Stower H. (2020). Lack of maternal-fetal SARS-CoV-2 transmission. Nat. Med. 26:312. 10.1038/s41591-020-0810-y 32161408 PMC7096028

[B80] Su S. Wong G. Shi W. Liu J. Lai A. C. K. Zhou J.. (2016). Epidemiology, genetic recombination, and pathogenesis of Coronaviruses. Trends Microbiol. 24, 490–502. 10.1016/j.tim.2016.03.003 27012512 PMC7125511

[B81] Tang W. Cao Z. Han M. Wang Z. Chen J. Sun W.. (2020). Hydroxychloroquine in patients with mainly mild to moderate coronavirus disease 2019: open label, randomised controlled trial. BMJ 369:m1849. 10.1136/bmj.m1849 32409561 PMC7221473

[B82] Temime L. Gustin M.P. Duval A. Buetti N. Crepey P. Guillemot D.. (2020). Estimating R0 OF SARS-COV-2 in healthcare settings. medRxiv 10.1101/2020.04.20.2007246232473007

[B83] To K. K. Tsang O. T. Chik-Yan Yip C. Chan K. H. Wu T. C. Chan J. M. C.. (2020). Consistent detection of 2019 novel coronavirus in saliva. Clin. Infect. Dis. 10.1093/cid/ciaa149 32047895 PMC7108139

[B84] Varga Z. Flammer A. J. Steiger P. Haberecker M. Andermatt R. Zinkernagel A. S.. (2020). Endothelial cell infection and endotheliitis in COVID-19. Lancet 395, 1417–1418. 10.1016/S0140-6736(20)30937-5 32325026 PMC7172722

[B85] Verdoni L. Mazza A. Gervasoni A. Martelli L. Ruggeri M. Ciuffreda M.. (2020). An outbreak of severe Kawasaki-like disease at the Italian epicentre of the SARS-CoV-2 epidemic: an observational cohort study. Lancet 10.1016/S0140-6736(20)31103-X 32410760 PMC7220177

[B86] Viner R. M. Whittaker E. (2020). Kawasaki-like disease: emerging complication during the COVID-19 pandemic. Lancet 10.1016/S0140-6736(20)31129-6 32410759 PMC7220168

[B87] Wahba L. Jain N. Fire A. Z. Shoura M. J. Artiles K. L. McCoy M. J.. (2020). Identification of a pangolin niche for a 2019-nCoV-like coronavirus through an extensive meta-metagenomic search. bioRxiv 10.1101/2020.02.08.93966032376697

[B88] Wang D. Hu B. Hu C. Zhu F. Liu X. Zhang J.. (2020). Clinical characteristics of 138 hospitalized patients with 2019 novel coronavirus-infected pneumonia in Wuhan, China. JAMA. 10.1001/jama.2020.1585 32031570 PMC7042881

[B89] Wang G. Zhang Y. Zhao J. Zhang J. Jiang F. (2020). Mitigate the effects of home confinement on children during the COVID-19 outbreak. Lancet 395, 945–947. 10.1016/S0140-6736(20)30547-X 32145186 PMC7124694

[B90] Wang H. Zhang L. (2020). Risk of COVID-19 for patients with cancer. Lancet Oncol. 21:e181 10.1016/S1470-2045(20)30149-2 32142621 PMC7129735

[B91] Wang L. Shi Y. Xiao T. Fu J. Feng X. Mu D.. (2020). Chinese expert consensus on the perinatal and neonatal management for the prevention and control of the 2019 novel coronavirus infection (First edition). Ann. Transl. Med. 8:47. 10.21037/atm.2020.02.20 32154287 PMC7036629

[B92] Wang M. Cao R. Zhang L. Yang X. Liu J. Xu M.. (2020). Remdesivir and chloroquine effectively inhibit the recently emerged novel coronavirus (2019-nCoV) *in vitro*. Cell Res. 30, 269–271. 10.1038/s41422-020-0282-0 32020029 PMC7054408

[B93] Wang Q. Zhang Y. Wu L. Niu S. Song C. Zhang Z.. (2020). Structural and Functional Basis of SARS-CoV-2 Entry by Using Human ACE2. Cell 181, 894–904.e899. 10.1016/j.cell.2020.03.045 32275855 PMC7144619

[B94] Wang W. Xu Y. Gao R. Lu R. Han K. Wu G.. (2020). Detection of SARS-CoV-2 in different types of clinical specimens. JAMA 323, 1843–1844. 10.1001/jama.2020.3786 32159775 PMC7066521

[B95] Wax R. S. Christian M. D. (2020). Practical recommendations for critical care and anesthesiology teams caring for novel coronavirus (2019-nCoV) patients. Can. J. Anaesth. 67, 565–578. 10.1007/s12630-020-01591-x 32052373 PMC7091420

[B96] Weiss S. R. Leibowitz J. L. (2011). “Chapter 4: Coronavirus Pathogenesis,” in Advances in Virus Research, eds K. Maramorosch, A. J. Shatkin, and F. A. Murphy (Philadelphia, PA: Academic Press), 85–164. 10.1016/B978-0-12-385885-6.00009-2 PMC7149603 22094080

[B97] Wilder-Smith A. Chiew C. J. Lee V. J. (2020). Can we contain the COVID-19 outbreak with the same measures as for SARS? Lancet Infect. Dis. 20, e102–e107. 10.1016/S1473-3099(20)30129-8 32145768 PMC7102636

[B98] Wilson N. Kvalsvig A. Barnard L. T. Baker M. G. (2020). Case-fatality risk estimates for COVID-19 calculated by using a lag time for fatality. Emerg. Infect. Dis. 26, 1339–1441. 10.3201/eid2606.200320 32168463 PMC7258483

[B99] Woo P. C. Huang Y. Lau S. K. Yuen K. Y. (2010). Coronavirus genomics and bioinformatics analysis. Viruses 2, 1804–1820. 10.3390/v2081803 21994708 PMC3185738

[B100] Working Group of 2019 Novel Coronavirus Peking Union Medical College Hospital. (2020). [Diagnosis and clinical management of 2019 novel coronavirus infection: an operational recommendation of Peking Union Medical College Hospital (V2.0)]. Zhonghua Nei Ke Za Zhi 59, 186–188. 10.3760/cma.j.issn.0578-1426.2020.03.003 32023681

[B101] Wrapp D. Wang N. Corbett K. S. Goldsmith J. A. Hsieh C. L. Abiona O.. (2020). Cryo-EM structure of the 2019-nCoV spike in the prefusion conformation. Science 367, 1260–1263. 10.1126/science.abb2507 32075877 PMC7164637

[B102] Wu C. Chen X. Cai Y. Xia J. Xu S. Zhou X.. (2020). Risk factors associated with acute respiratory distress syndrome and death in patients with coronavirus disease 2019 pneumonia in Wuhan, China. JAMA Intern. Med. E200994. 32167524 10.1001/jamainternmed.2020.0994 PMC7070509

[B103] Wu F. Zhao S. Yu B. Chen Y. Wang W. Song Z.G.. (2020). A new coronavirus associated with human respiratory disease in China. Nature 579, 1–8. 10.1038/s41586-020-2008-3 32015508 PMC7094943

[B104] Wu J. T. Leung K. Leung G. M. (2020). Nowcasting and forecasting the potential domestic and international spread of the 2019-nCoV outbreak originating in Wuhan, China: a modelling study. Lancet 395, 689–697. 10.1016/S0140-6736(20)30260-9 32014114 PMC7159271

[B105] Xia Y. Jin R. Zhao J. Li W. Shen H. (2020). Risk of COVID-19 for cancer patients. Lancet Oncol. 21:E181. 32142622 10.1016/S1470-2045(20)30150-9 PMC7130057

[B106] Xiao K. Zhai J. Feng Y. Zhou N. Zhang X. Zou J.J. (2020). Isolation and characterization of 2019-nCoV-like coronavirus from malayan pangolins. bioRxiv 10.1101/2020.02.17.951335

[B107] Xiao Y. Torok M. E. (2020). Taking the right measures to control COVID-19. Lancet Infect. Dis. 20, 523–524. 10.1016/S1473-3099(20)30152-3 32145766 PMC7128408

[B108] Xu Z. Shi L. Wang Y. Zhang J. Huang L. Zhang C.. (2020). Pathological findings of COVID-19 associated with acute respiratory distress syndrome. Lancet Respir. Med. 8, 420–422. 10.1016/S2213-2600(20)30076-X 32085846 PMC7164771

[B109] Yin W. Mao C. Luan X. Shen D. D. Shen Q. Su H.. (2020). Structural basis for inhibition of the RNA-dependent RNA polymerase from SARS-CoV-2 by remdesivir. Science abc1560. 10.1126/science.abc1560 32358203 PMC7199908

[B110] Yuan M. Wu N. C. Zhu X. Lee C. D. So R. T. Y. Lv H.. (2020). A highly conserved cryptic epitope in the receptor binding domains of SARS-CoV-2 and SARS-CoV. Science 368, 630–633. 10.1126/science.abb7269 32245784 PMC7164391

[B111] Zandifar A. Badrfam R. (2020). Iranian mental health during the COVID-19 epidemic. Asian J. Psychiatr 51:101990. 10.1016/j.ajp.2020.101990 32163908 PMC7128485

[B112] Zhang H. Zhou P. Wei Y. Yue H. Wang Y. Hu M.. (2020). Histopathologic changes and SARS–CoV-2 immunostaining in the Lung of a patient With COVID-19. Ann. Intern. Med. 172, 629–632. 10.7326/M20-0533 32163542 PMC7081173

[B113] Zhao J. P. Hu Y. Du R. H. Chen Z. S. Jin Y. Zhou M. (2020). [Expert consensus on the use of corticosteroid in patients with 2019-nCoV pneumonia]. *Zhonghua Jie He He Hu Xi Za Zhi* 43:E007. 10.3760/cma.j.issn.1001-0939.2020.0007 32034899

[B114] Zhou F. Yu T. Du R. Fan G. Liu Y. Liu Z.. (2020). Clinical course and risk factors for mortality of adult inpatients with COVID-19 in Wuhan, China: a retrospective cohort study. Lancet 395, 1054–1062. 10.1016/S0140-6736(20)30566-3 32171076 PMC7270627

[B115] Zhou P. Yang X. L. Wang X. G. Hu B. Zhang L. Zhang W.. (2020). A pneumonia outbreak associated with a new coronavirus of probable bat origin. Nature 579, 270–273. 10.1038/s41586-020-2012-7 32015507 PMC7095418

[B116] Zhou Y. Fu B. Zheng X. Wang D. Zhao C. Qi Y.. (2020). Pathogenic T cells and inflammatory monocytes incite inflammatory storm in severe COVID-19 patients. Natl. Sci. Rev. 10.1093/nsr/nwaa041 34676125 PMC7108005

[B117] Zhu N. Zhang D. Wang W. Li X. Yang B. Song J.. (2020). A novel coronavirus from patients with pneumonia in China, 2019. N. Engl. J. Med. 382, 727–733. 10.1056/NEJMoa2001017 31978945 PMC7092803

